# Distinct plasma lipids profiles of recurrent ovarian cancer by liquid chromatography-mass spectrometry

**DOI:** 10.18632/oncotarget.11603

**Published:** 2016-08-25

**Authors:** Junnan Li, Hongyu Xie, Ang Li, Jinlong Cheng, Kai Yang, Jingtao Wang, Wenjie Wang, Fan Zhang, Zhenzi Li, Harman S. Dhillon, Margarita S Openkova, Xiaohua Zhou, Kang Li, Yan Hou

**Affiliations:** ^1^ Department of Epidemiology and Biostatistics, School of Public Health, Harbin Medical University, Harbin 150086, P.R. China; ^2^ Department of Gynecology Oncology, The Affiliated Tumor Hospital of Harbin Medical University, Harbin 150086, P.R. China; ^3^ Harbin Medical University, Harbin 150086, P.R. China; ^4^ Department of Biostatistics, University of Washington, Seattle 96596, WA, U.S.A; ^5^ Key Laboratory of Cardiovascular Medicine Research (Harbin Medical University), Ministry of Education, Harbin 150086, P.R. China

**Keywords:** epithelial ovarian cancer, lipidomics, recurrence, early recurrence

## Abstract

Epithelial ovarian cancer (EOC) is the most deadly gynecologic malignancy worldwide due to its high recurrence rate after surgery and chemotherapy. There is a critical need for discovery of novel biomarkers for EOC recurrence providing higher prediction power than that of the present ones. Lipids have been reported to associate with development and progression of cancer. In the current study, we aim to identify and validate the lipids which were relevant to the ovarian cancer recurrence based on plasma lipidomics performed by ultra-performance liquid chromatography coupled with mass spectrometry. In order to fulfill this objective, plasma from 70 EOC patients with follow up information was obtained. The results revealed that patients with and without recurrence could be clearly distinguished based on their lipid profiles. Thirty-one lipid metabolites were identified as potential biomarkers for EOC recurrence. The AUC value of these metabolite combinations for predicting EOC recurrence was 0.897. In terms of clinical applicability, LysoPG(20:5) arose as a potential EOC recurrence predictive biomarker to increase the predictive power of clinical predictors from AUC value 0.739 to 0.875. Additionally, we still found that individuals with early relapses (< 6 months) had a distinctive metabolomic pattern compared with late EOC and non-EOC recurrence subjects. Interestingly, decreased levels of triglycerides (TGs) were found to be a specific metabolic feature foreshadowing an early relapse. In conclusion, plasma lipidomics study could be used for predicting EOC recurrences, as well as early and late recurrent cases. The lipid biomarker research improves the predictive power of clinical predictors and the identified biomarkers are of great prognostic and therapeutic potential.

## INTRODUCTION

Epithelial ovarian cancer (EOC) is the most fatal gynecologic malignancy worldwide. Approximately 204,000 new EOC cases are diagnosed each year, of which 125,000 women would die annually [1]. Cytoreductive surgery followed by platinum-based therapy combined with paclitaxel become the standard therapy in EOC [2, 3]. Despite ongoing progress in cytoreductive surgery and high initial chemotherapy sensitivity, more than 60% of patients with advanced stages will still relapse within five years after treatment. These patients are rarely curable and have a 5-year overall survival rate of 25–35% [4, 5]. Among the patients with a high risk of recurrence, early prediction may provide novel therapeutic modalities to improve their clinical outcomes and enhance survival rate. Though studies have concentrated on the prognostic importance of conventional demographics and clinical pathological predictors (such as age, FIGO stage, and grade in ovarian cancer), the variability in progression-free and overall survival rate differs among patients with similar clinical and pathological characteristics, which makes it difficult to foresee the outcome reliably [6–8]. Recently, more studies have been focusing on identifying new genes, or protein markers to predict recurrence in EOC patients, but such markers might be costly [9, 10]. Thus, there is a great need for discovering effective and accessible predictive markers for EOC recurrence. Such biomarkers would facilitate implementation of both second-line chemotherapy and molecular targeted therapy.

Lipids, as a kind of vital metabolites, comprise the majority of cellular membranes and involve in several cellular functions, including energy storage, cell differentiation and cell signaling. Over the past decade, numerous studies have demonstrated that dysregulated lipid metabolism was associated with the diagnosis and pathogenesis of human cancers, such as pancreatic adenocarcinoma, colon cancer, hepatocellular carcinoma, glioblastoma and prostate cancer [11–15]. Our previous metabolomic studies have demonstrated that lysophosphatidylcholine (LysoPC) might be a potential asset in the discrimination between EOC and controls [16]. In addition, we have previously reported a lipidomics study based on global plasma lipid profiling and found that a series of glycerophospholipids (GPs) were decreased, while a series of sphingolipids (SPs) were increased in EOC patients [17]. Besides, several studies have identified the predictive lipid biomarkers for disease recurrence [18, 19]. Allott *et al*. reported that higher levels of total cholesterol and triglycerides, in the blood of men who underwent surgery for prostate cancer, were associated with an increased risk for disease recurrence [20]. MarionaJové *et al*. showed that lipids could be used to predict stroke relapse, and found that low concentrations of a specific LysoPC were significantly associated with stroke recurrence [21]. In particular, our previous study performed a plasma metabolic profiling to predict recurrence of advanced EOC, and found that LysoPC and Lysophosphatidylethanolamine (LysoPE) were potential biomarkers [22]. However, plasma lipid profiling has not been performed to investigate EOC prognostic biomarkers systematically. Their potential role in EOC progression is currently under investigation.

In the present study, we employed an ultra-performance liquid chromatography coupled with mass spectrometry (UPLC/MS) lipid profiling strategy to plasma samples obtained from 70 EOC patients. Our goals were to determine if lipidomics could (i) discriminate EOC recurrent patients from non-recurrent ones, as well as distinguish between early and late relapse, (ii) discover potential lipid prognostic biomarkers, and (iii) identify the temporal pattern of EOC recurrence.

## RESULTS

### Demographics and clinic pathological characteristics of patients

A total of 70 patients were enrolled into the study. The median follow up time was 49 months. Table 1 displayed the baseline characteristics of all groups, comprising 39 EOC recurrent patients (12 ER patients and 27 LR patients) and 31 NR patients together. Serum CA-125 level, omentum metastasis, FIGO stage, histological differentiation, and lymph node metastasis were significantly different between recurrent and non-recurrent groups (*P* < 0.05). Noteworthy, no difference was found between ER and LR groups, regarding the listed characteristics.

**Table 1:** Detailed demographic and clinical characteristics of EOC patients

Characteristics	Recurrence (*N* = 39) (%)			Non-recurrence (*N* = 31) (%)	*P*-value
	ER (*N* = 12)	LR (*N* = 27)	Total		
Age					
< 50	7(58.33)	8(29.63)	15(38.46)	12(38.71)	0.9831
≥ 50	5(41.67)	19(70.37)	24(61.54)	19(61.29)	
Serum CA-125 level					
< 35	1(8.33)	0(0)	1(2.56)	6(19.35)	0.0240*
≥ 35	11(91.67)	27(100)	38(97.44)	25(80.65)	
Greater Omentum metastasis					
Absent	3(25.00)	5(18.52)	8(20.51)	19(61.29)	0.0007*
Present	8(66.67)	20(74.07)	28(71.79)	11(35.48)	
Undocumented	1(8.33)	2(7.41)	3(7.70)	1(3.23)	
FIGO stage					
I	1(8.33)	1(3.70)	2(5.13)	12(38.71)	0.0033*
II	0(0)	3(11.11)	3(7.69)	3(9.68)	
III	10(83.34)	22(81.48)	32(82.05)	16(51.61)	
IV	1(8.33)	1(3.71)	2(5.13)	0(0)	
Histology differentiation					
Well	0(0)	0(0)	0(0)	8(25.80)	0.0015*
Moderately	3(25.00)	2(7.41)	5(12.82)	5(16.13)	
Poorly	9(75.00)	21(77.78)	30(76.92)	14(45.16)	
Undocumented	0(0)	4(14.81)	4(10.26)	4(12.90)	
Lymph node metastasis					
Absent	7(58.33)	18(66.67)	25(64.10)	29(93.55)	0.0036*
Present	5(41.67)	9(33.33)	14(25.90)	2(6.55)	

* statistically significance between recurrence in total and non-recurrence group;

Percentage in brackets is the column percent.

### Distinctive plasma lipid profiles of recurrent and non-recurrent EOC

**Figure 1:**
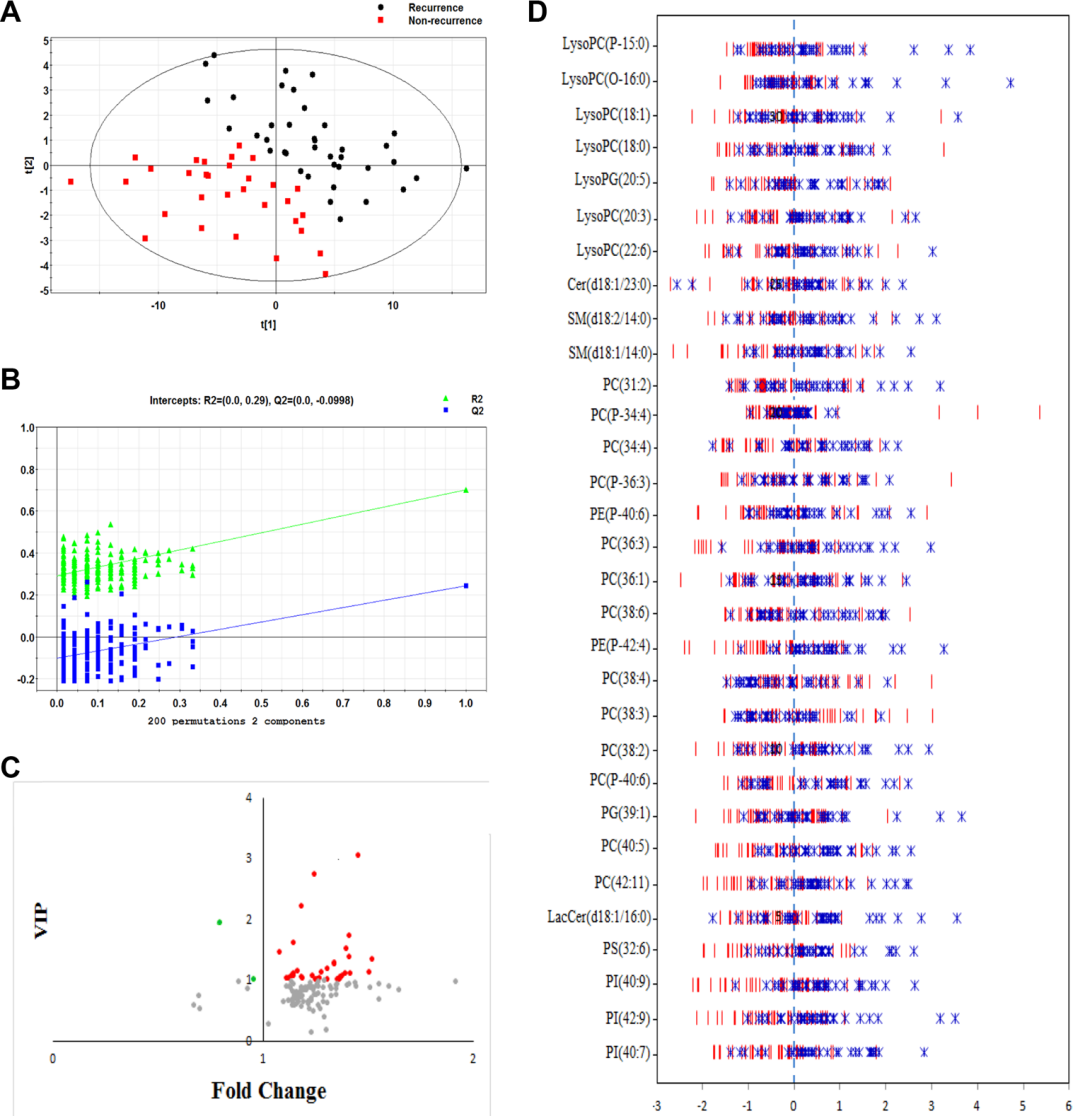
(**A**) PLS-DA score plot distinguishing EOC recurrence from non-EOC recurrence (two latent variables with the performance of R2X = 0.364, R2Y = 0.701 and Q2 = 0.224); (**B**) Validation plot for discriminating between EOC recurrence and non-EOC recurrence with 200 permutations; (**C**) The scatter plot depicting the importance of metabolites in discriminating EOC recurrence from non-EOC recurrence; red dots represent down-regulation in EOC patients with recurrence, green dots represent up-regulation in EOC patients with recurrence; (**D**) The Z-score plot of differentiating metabolites between EOC recurrence and non-EOC recurrence. The values were standardized using mean centering and unit variance scaling of each variable.

After the peak alignment and removal of isotopic peaks and adducts, as described in previous publications, 459 ions were measured in the ESI+ model [17]. PCA score plots revealed that the QC samples were tightly clustered, indicating the robustness of our metabolic profiling platform (Supplementary Figure S1). However, the unsupervised PCA did not detect obvious separation trends between those with and without recurrence. All of the statistically significant ions (Kruskal–Wallis rank sum test, *P* < 0.05) were subjected to further analysis. A supervised PLS-DA model was used to distinguish the difference between plasma samples in patients with and without EOC recurrence. The PLS-DA score revealed a clear separation between groups, which indicated that recurrent and non-recurrent EOC had different lipid profiling (Figure 1A). The cumulative R2Y and Q2 were 0.701 and 0.224, respectively, when two principal components were calculated. Permutation test with 200 iterations was performed to avoid the overfitting, and no overfitting was observed (Figure 1B).

### Important lipid alterations related to recurrent EOC

Following VIP values with a threshold of 1.0, differential lipids were selected as potential biomarkers for subsequent identification. Structure identification was performed, as described in our previous study [17]. In total, 31 differential lipids were selected as potential lipid biomarkers of recurrent EOC, which were presented in Figure 1C and 1D and also listed in Table 2. Most of the identified lipids in EOC recurrent patients were decreased compared with the non-recurrent ones, except up-regulated PC(31:2) and PE-P(42:4) in EOC recurrent patients. The detailed individual data of these lipids between two groups were shown in Supplementary Figure S2. Mapping these differential metabolites to their biochemical pathways through database searches (HMDB, KEGG, and Lipidmaps) revealed evident disorders in the sphingolipid signaling pathway and glycerophospholipid metabolism.

**Table 2:** Identified differential metabolites between with and without recurrent EOC

ID	Lipid	MZ	rt (min)	ppm	FC	*P*-value	VIP	AUC
1	LysoPC(P-15:0)	466.3296	4.54	0.77	0.66	1.50E-05	1.34	0.792
2	LysoPC(O-16:0)	482.3592	2.91	2.60	0.70	0.0001	1.11	0.759
3	LysoPC(18:1)	522.3542	2.52	2.21	0.72	2.90E-05	1.08	0.783
4	LysoPC(18:0)	524.3717	3.85	1.06	0.74	0.0002	1.01	0.754
5	LysoPG(20:5)	531.2742	1.64	4.53	0.69	0.0006	3.05	0.736
6	LysoPC(20:3)	546.353	3.85	4.48	0.73	0.0001	1.01	0.763
7	LysoPC(22:6)	568.3387	2.08	1.96	0.71	1.22E-06	1.39	0.824
8	Cer(d18:1/23:0)	636.6274	18.58	2.28	0.81	0.0137	1.08	0.672
9	SM(d18:2/14:0)	673.5263	10.91	2.43	0.73	0.0024	1.05	0.71
10	SM(d18:1/14:0)	675.5436	12.62	0.02	0.76	0.0002	1.01	0.757
11	PC(31:2)	716.5254	15.14	4.09	1.25	0.0290	1.94	0.653
12	PC(P-34:4)	738.5461	13.25	3.90	0.71	3.92E-06	1.52	0.81
13	PC(34:4)	754.5385	13.55	0.41	0.74	0.0308	1.28	0.651
14	PC(P-36:3)	768.591	15.36	1.05	0.88	0.0256	1.08	0.656
15	PE(P-40:6)	776.5617	16.16	3.55	0.71	0.0017	1.73	0.717
16	PC(36:3)	784.5864	16.45	1.60	0.93	0.0403	1.45	0.644
17	PC(36:1)	788.6185	16.53	2.61	0.87	0.0147	1.11	0.67
18	PC(38:6)	806.5688	13.98	0.87	0.79	0.0025	1.03	0.709
19	PE(P-42:4)	808.624	16.94	3.09	1.04	0.0358	1.01	0.647
20	PC(38:4)	810.6009	16.53	0.06	0.87	0.0137	1.08	0.672
21	PC(38:3)	812.6174	16.11	1.20	0.80	0.0256	1.01	0.656
22	PC(38:2)	814.6357	16.69	4.42	0.79	0.0039	1.01	0.7
23	PC(P-40:6)	818.6053	14.89	0.60	0.86	0.0358	1.14	0.647
24	PG(39:1)	819.6087	16.32	2.80	0.89	0.0465	1.04	0.639
25	PC(40:5)	836.615	16.70	1.66	0.87	0.0174	1.61	0.666
26	PC(42:11)	852.5512	13.84	3.00	0.78	0.0008	1.13	0.731
27	LacCer(d18:1/16:0)	862.6208	14.26	4.91	0.84	0.0060	1.05	0.691
28	PS(32:6)	878.5878	16.54	3.23	0.90	0.0218	1.04	0.66
29	PI(40:9)	905.5171	13.60	0.46	0.84	0.0186	1.03	0.664
30	PI(42:9)	909.5477	14.75	1.21	0.66	0.0097	1.12	0.68
31	PI(40:7)	933.5453	14.62	3.71	0.74	0.0021	1.27	0.712

MZ mass-to-charge ratio, rt retention time, ppm parts per million, FC fold change, VIP variable important in projection.

### Predictive performance of recurrent EOC biomarkers

**Figure 2:**
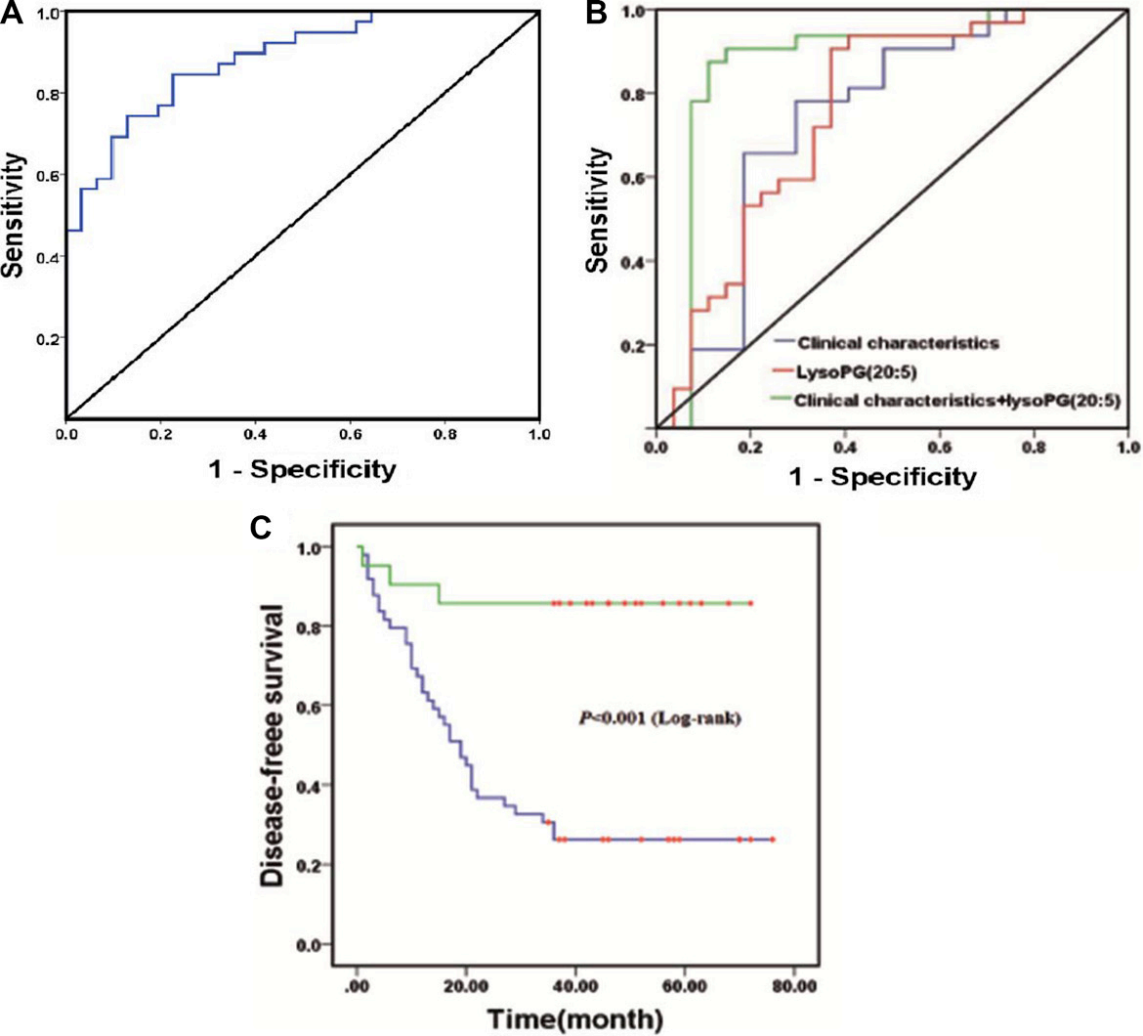
(**A**) ROC curves based on the random forest model with leave-one-out cross-validation for prediction with 31 candidate lipid biomarkers; (**B**) The inclusion of LysoPG(20:5) level to related prognostic clinical characteristics including serum CA-125 level, omentum metastasis, FIGO stage, histology differentiation grade and lymph node metastasis to receiver operating characteristic curve increase the predictive power of EOC recurrence (area: clinical characteristics: 0.739, *P* < 0.01(blue line); LysoPG(20:5): 0.736, *P* < 0.001 (red line); clinical characteristics + LysoPG(20:5): 0.875, *P* < 0.001(green line)); (**C**) Kaplan–Meier curve comparing EOC recurrence with lower LysoPG(20:5) values (blue line) and higher LysoPG(20:5) values (green line).

In order to evaluate the capacity of these potential biomarkers, a random forest based on leave-one out cross-validation and receiver operating characteristic (ROC) curves were performed. As expected, the panel of 31 metabolites was able to discriminate between patients with and without recurrent EOC, with an AUC value of 0.897 (Figure 2A), which suggests strong potential for predicting EOC recurrence. However, it is impossible to predict recurrence with many biomarkers in clinical practice. Therefore, identifying a few biomarkers or a panel of biomarkers, which could provide greater predictive ability, was particularly important. A binary logistic regression analysis carried out recurrence results as the dependent variables (0 = non-recurrence and 1 = recurrence) and these candidate lipids and related prognostic clinical characteristics (including omentum metastasis, FIGO stage, histological differentiation, and lymph node metastasis; see Supplementary Table S1) as the independent variables. As a result, LysoPG(20:5), as a potential biomarker, could provide an AUC value of 0.736, significantly increasing the predictive power of clinical characteristics from AUC value 0.739 to 0.875 (Figure 2B). The mass spectra and the possible fragment structures were then performed to confirm the chemical structures of the LysoPG(20:5), which were shown in Supplementary Figure S3 and Supplementary Figure S4 Based on the Youden index J = max (sensitivity +specificity-1), a cut-off value of LysoPG (20:5) was selected. Seventy patients were divided into two groups based on the selected cut-off value. A Kaplan-Meier was then performed for the recurrence rate analysis. The overall time to recurrence for recurrence in those patients with predicted probability below the cut-off value, was significantly lower than those with LysoPG(20:5) value above the cut-off value (30 vs 62 months) (Figure 2C).

### Patients with early recurrent EOC have a specific metabolomic pattern

**Figure 3:**
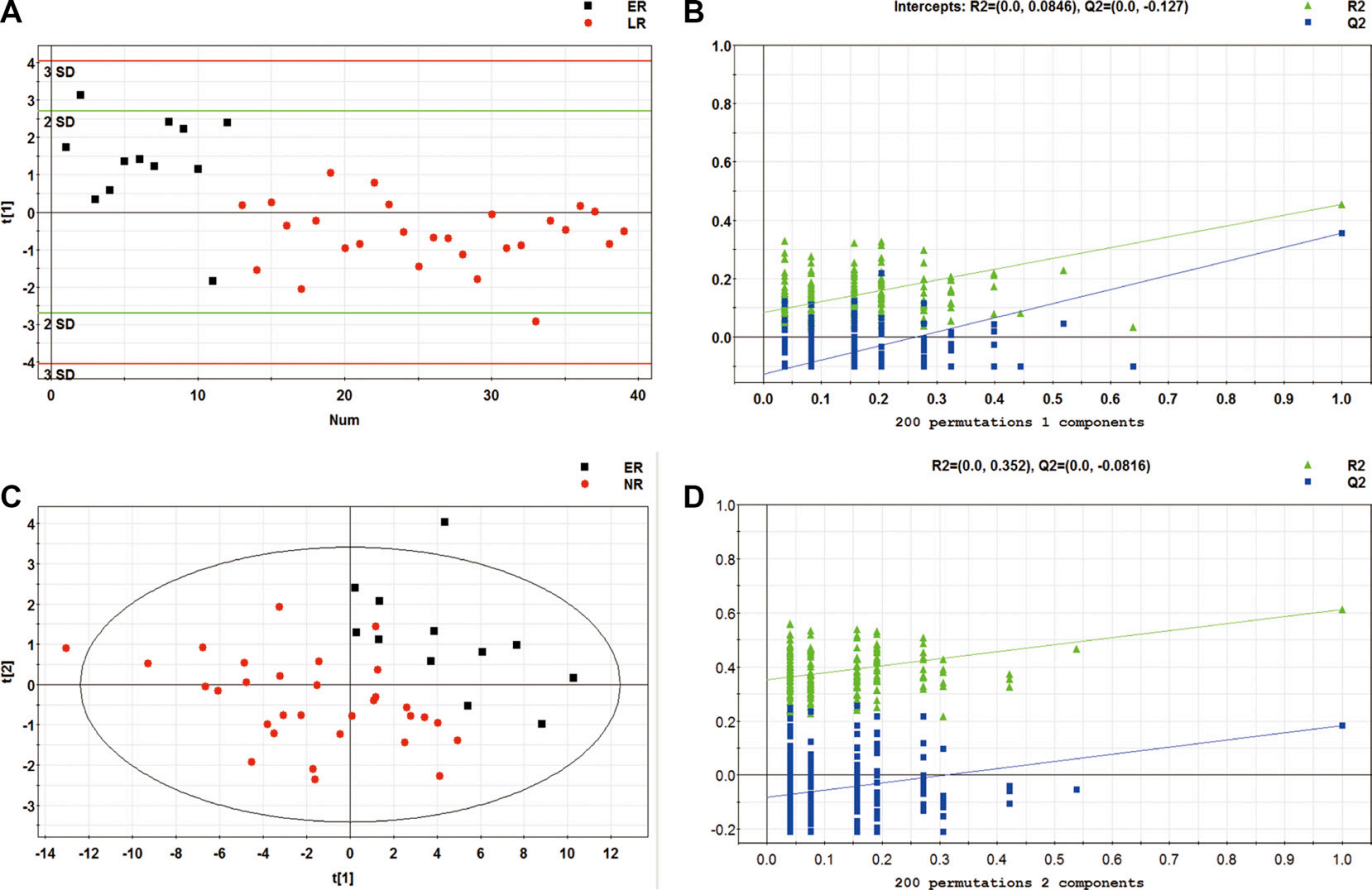
(**A**) PLS-DA score plot for discriminating early and late EOC recurrence; (**B**) Validation plot for discriminating early and late recurrent EOC patients with 200 permutations; (**C**) PLS-DA score plot for discriminating early and non-EOC recurrence; (**D**) Validation plot for discriminating early and non-EOC recurrence. ER: early recurrence; LR: late recurrence; NR: non-recurrence.

We focused on early recurrent EOC because of its challenging clinical situation. We evaluated metabolomic profile differences between early and late relapses, as well as between early and non-relapse patients. EOC patients in early relapse were distinguishable from those in late relapse, with an AUC value of 0.756 (Figure 3A and 3B). Likewise, the discrimination between early relapse and non-recurrent patients was robust, with an AUC value of 0.884 (Figure 3C and 3D). The results revealed that metabolomic profiles were able to offer a highly accurate prediction of early relapse.

### Temporal patterns of differential metabolites related to recurrent EOC

**Figure 4:**
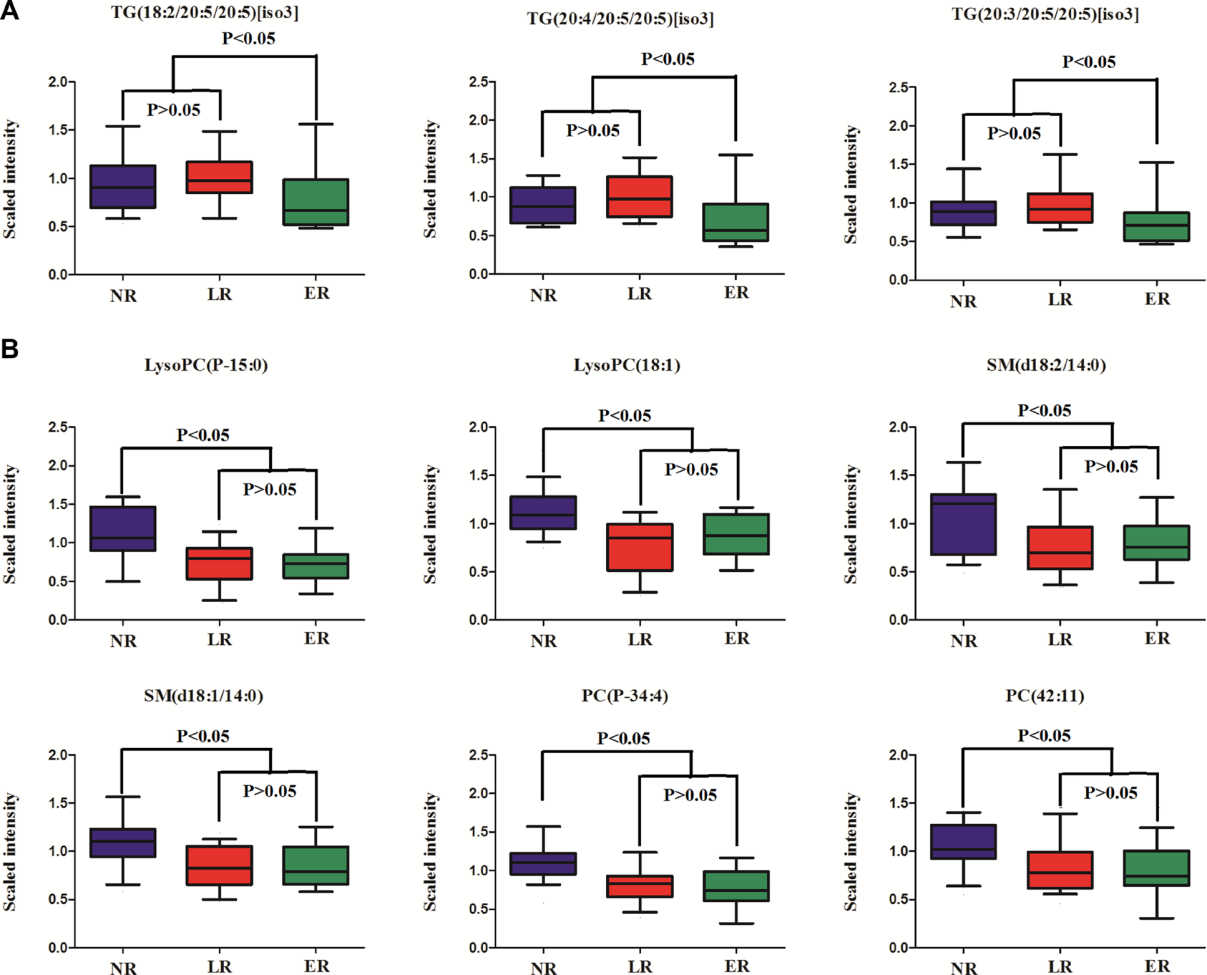
Changing patterns of differential metabolites from non-EOC recurrence across late recurrence and early recurrence (**A**) Significant alterations in early recurrence compared to late relapse and non-recurrence, but no difference among late and non-recurrence; (**B**) Significant alterations in non-recurrence compared to early and late recurrence, but no difference among early and late recurrence. ER: early recurrence; LR: late recurrence; NR: non-recurrence.

To further investigate the potential dynamics of the differential metabolites during EOC progression, we compared metabolite patterns of NR to LR and ER. In summary, there are two categories of metabolites. One represents significant alterations in early relapse compared to late relapse and non-recurrence, but no difference among late relapse and non-recurrence (Figure 4A). The other shows significant alterations in non-recurrence compared to early and late relapses, but no difference among early and late relapses (Figure 4B). Unfortunately, we found that no metabolite had significant alterations among early relapse, late relapse and non-relapse. Remarkably, three triglycerides (TGs) were dysregulated in ER patients, which deserves our attentions. Unexpectedly, the plasma concentrations of many LysoPCs, phosphatidylcholine (PCs) and phosphatidylinositols (PIs) were up-regulated in LR patients in comparison to ER patients, whereas, they were remarkably low in non-EOC patients.

## DISCUSSION

Recent evidence indicated that cancer progression was usually associated with changes in the levels of different lipid species, because lipid production has been linked to an increased need for membranes during rapid cell proliferation and apoptosis [23]. In this study, a non-targeted lipidomics approach using UPLC-MS has been applied to fingerprint the plasma lipids to screen for potential biomarkers of EOC recurrence. Our study suggested that the plasma lipidomics analyzed by UPLC-QTOF/MS could be used to discriminate EOC recurrence from non-recurrence, and the 31 differential metabolites were highly associated with EOC recurrence. In addition, the lipid changes were found between early and late EOC recurrence, as well as between early and non-EOC recurrence. Meanwhile, the ascertained biomarker significantly improved prediction capacities of related prognostic clinical characteristics, such as CA-125 level, omentum metastasis, FIGO stage, histological differentiation grade, and lymph node metastasis. The excellent prediction performance through the plasma lipids suggests that the lipidomics approach might be particularly noteworthy, especially for predicting EOC recurrence remains challenging around the world, especially in early EOC relapse. These findings support the hypothesis that tumor molecular characteristics may predict the survival outcome, in addition to clinical characteristics.

There is a considerable interest in developing a prognostic model for ovarian cancer, which can be used for predicting the risk of EOC recurrence in clinical practice. There have been several attempts to design clinically feasible prognostic models in ovarian cancer. Liu *et al*. developed a prognostic model for disease-free survival (PFS) in 161 primary EOC patients and found that FIGO stage, histological grade, residual disease after primary surgery, recurrent season, and adjuvant chemotherapy cycles were associated with a significantly greater risk of recurrence [24]. Hendrickson *et al*. developed a prognostic model for 12-month PFS derived from Mayo Clinic Ovarian Cancer registry, and found that histological subtype, grade, CA-125 and stage were strongly associated with PFS [25]. However, age at diagnosis and CA125 pre-surgery were not significantly associated with PFS. In contrast, we found that omentum metastasis and lymph node metastasis were associated with PFS. Particularly, decreased LysoPG(20:5) level was identified as the most important prognostic feature in our model, which may provide additional prognostic information for EOC. LysoPG stimulated intracellular calcium signaling via phospholipase C activation in OVCAR-3 human ovarian cancer cells [26]. It also stimulated chemotactic migration in endothelial cells of human umbilical vein and human natural killer cells in a PTX-sensitive manner. These cellular responses support the assumption that LysoPG acts as a lysophospholipid mediator through possible G protein–coupled receptors (GPCRs) for LysoPG. GPCRs could result in the initiation of tumor growth and survival pathways, and may, therefore, play important roles in the development and progression of EOC. However, such specific GPCRs have not been elucidated so far. It is also suggested that LysoPG behaves as a lysophosphatidic acid (LPA) receptor antagonist.

Consistent with our previous metabolomics-based reports on EOC recurrence, LysoPCs were down-regulated in recurrent EOC patients compared with the non-recurrent patients [22]. It has been reported that LysoPC is an important signaling molecule in regulating cellular proliferation, inflammation, and cancer cell invasion. It is also the substrate of lysophospholipase D, an enzyme converting LysoPC to LPA during cancer progression [27]. Lysophospholipase D over-expression has been determined in several cancers, such as breast cancer, glioblastoma, and hepatocellular carcinoma [28, 29]. A recent study has also suggested that decreased LysoPC (16:0) raises the risk of stroke recurrence [21]. Thus, alterations in LysoPCs metabolism may, therefore, play important roles in the development and progression of EOC.

PC is an important constituent of the choline-containing metabolite signaling. It is involved in cell signaling, structural integrity of the cell membrane, and is a breakdown product of the catabolic pathway of choline phospholipid metabolism, which may serve as an index of membrane change. In this study, a series of PCs were down-regulated in EOC recurrent patients, which suggests a higher cellular proliferation rate of ovarian cancer cells in recurrent patients. Altered PC metabolism was found in breast and prostate cancer cell lines, and a previous study suggested that abnormal PC metabolism in EOC could provide choline-based imaging approaches as powerful tools, to improve diagnosis and identify new therapeutic targets [30]. In current study, the decreased plasmenylcholine (pPCs) and plasmenylethanolamine (pPEs) levels in EOC recurrent patients suggested that most cancer cells might exhibit elevated oxidative stress, which is consistent with previous findings that oxidative stress is associated with cancer progression.

Three sphingolipids, Cer(d18:1/23:0), SM(d18:1/14:0), SM(d18:2/14:0) were decreased in EOC recurrent patients. Bioactive sphingolipid metabolites have served as important lipid second messengers in the regulation of tumor cell survival, cell growth, differentiation, migration and angiogenesis [31]. Ceramide is metabolized by ceramide kinase to generate ceramide-1-phosphate (C1P) and by ceramidase to generate sphingosine, which is further phosphorylated to sphingosine-1-phosphate (S1P) by sphingosine kinase. High ceramide kinase expression has been reported to be associated with poor recurrence-free survival in women with ER-negative breast cancer [32]. S1P and sphingosine kinase, have been implicated in many cellular processes including cell growth, proliferation, survival, and migration. These findings suggest that defects on ceramide generation and sphingolipid metabolism exist in order to promote cancer cell survival and cancer therapy resistance. Altogether, these opinions pinpoint the evidence that ceramide plays a role in cancer, as well as identify a potential target for the treatment and prevention of ovarian cancer recurrence.

In this study, PIs levels were lower in patients with recurrent EOC than in those without recurrent EOC. PIs were metabolized by 1-phosphatidylinositol-3-phosphate 5-kinase (PIKfyve) to generate phosphatidylinositol 5-phosphate, which was further metabolized by 1-phosphatidylinositol-5-phosphate 4-kinase (PIP4K2) to generate phosphatidylinositol-4,5-bisphosphate (PI(4,5)P2) [33] 05:30. PI(4,5)P2 was the direct substrate of phosphatidylinositol 3-kinase (PI3K)/AKT signaling pathway conducted by PIK3C to generate PI(3,4,5)P3. Recent studies have shown that the PI3K/AKT pathway was frequently disturbed in ovarian cancer, and had a vital role in the resistance of ovarian cancer cells to cisplatin and recurrence of ovarian cancer [34, 35].

**Figure 5:**
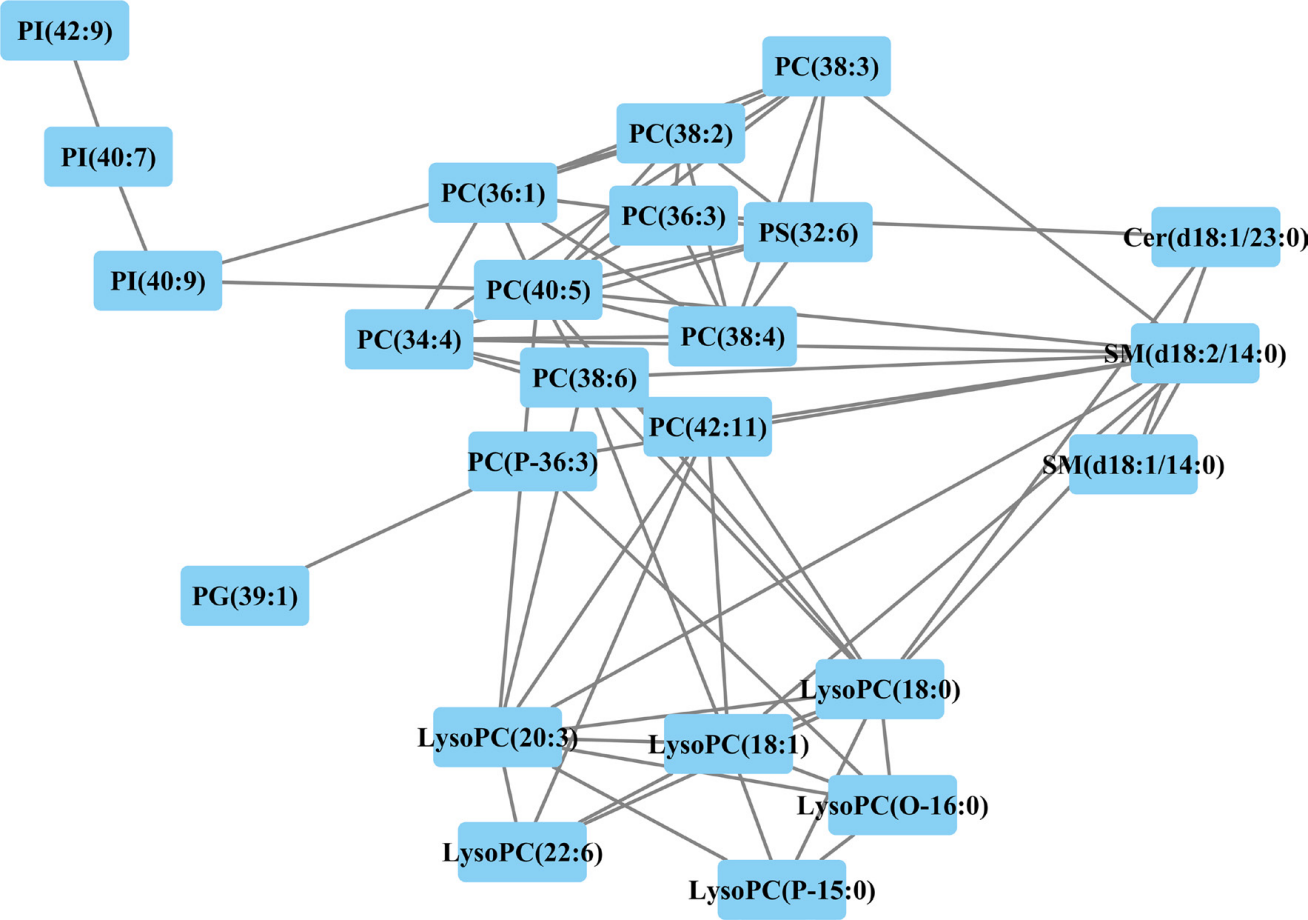
Correlation network of differential lipids between patients with and without EOC recurrence related metabolites (Pearson correlation analysis, |*r*| > 0.6) are connected with a line

A correlation network based on the Pearson correlation coefficients was constructed using Cytoscape software to explore the latent relationships of differential lipid species (Figure 5). A total of 24 differential lipids were included in this network, while the other seven lipids were excluded because of |*r*| < 0.6. A remarkable connection between LysoPCs, PCs, and SPs suggested that a series of biological conversions between GPs and SPs might occur during EOC recurrence. PS was located between PCs and Cer, which might reflect the fact that PCs and Cer can be transformed between each other by PS, given that PS participates in ceramide biosynthesis as serine donors. Labeling of ceramide by serine from PS provides evidence for a new metabolic relationship between GPs and SPs. PIs were also associated with LysoPCs, possibly suggesting that LysoPCs interact with them to exert effects. This figure reinforces the fact that each subclass was tightly correlated.

Interestingly, lower plasma levels of serum TGs arose as potential predictor of early EOC recurrence. TG, as an important energy storage form, is closely related to glucose homeostasis and its dysregulation is associated with the onset of metabolic syndrome such as diabetes, obesity, and cardiovascular diseases [36]. The three types of fatty acids that compose triglycerides include: saturated, monounsaturated and polyunsaturated fatty acids (PUFAs). PUFAs are essential, as they are biologically active molecules that serve as structural components of cellular membranes and play important roles in metabolism, inflammation, cell signaling, and regulating gene expression. A large cohort, conducted by Murff *et al*., reported women with lower intake of long chain n-3 PUFAs and higher intake of n-6 PUFA had an increased risk for breast cancer compared with women with higher intake of long chain n-3 PUFAs and lower intake of n-6 PUFAs [37]. Another study, comprising of 30,252 breast cancer patients, revealed that eicosapentaenoic acid and docosahexaenoic acid were significantly inversely associated with risk for breast cancer (HR: 0.70, 95% CI: 0.54–0.90; HR: 0.67, 95% CI: 0.52–0.87) [38]. A recent study showed that PUFAs decreased TG levels in plasma [39]. In addition, Mika Hilvo *et al*. indicated that monounsaturated fatty acids in serum triacylglycerols were associated with response to neoadjuvant chemotherapy in breast cancer patients [40]. However, Allott *et al*. reported that higher levels of triglycerides the blood of male patients were associated with increased risk for prostate cancer recurrence, which presented a reverse trend to that in our study [20]. Overall, a trend of down-regulation of TGs in current study may originate from the accumulation of PUFAs, which might be correlated with chemotherapy-resistance, leading to the early relapse in EOC patients.

In terms of limitations of the present work, we recognize small sample size for EOC recurrence, especially early recurrence, among the studied population, which might exhibit a considerable variance in certain metabolites. Future studies with larger independent cohorts are necessary to validate the current findings. Furthermore, plasma specimens were collected upon initial tumor presentation from EOC patients, without controlling previous food intake, as well as the lack of blood samples after surgery. Moreover, we were not able to identify an important percentage of lipids present in samples, due to the major bottle neck in lipidomics: the lack of comprehensive lipid databases.

In summary, we have presented a holistic view of the plasma lipid changes related to EOC recurrence and temporal pattern. Lipid metabolites of EOC recurrent patients, as expected, differ a lot from that of non-EOC recurrent patients, and potential lipid markers have been identified that could be used to predicted EOC recurrence. Extending our previous lipid studies on EOC, the current study provided comprehensive lipid changes on the recurrence of EOC. At last, we have described the lipid metabolic characteristics associated with early recurrence, which might provide additional information concerning the recurrent mechanism of EOC that allows us to expand our understanding of EOC progression, potentially facilitate the medical management, and improve clinical outcomes.

## MATERIALS AND METHODS

### Sample collection and treatment

This study was approved by the Tumor Hospital Institutional Review Board of Harbin Medical University. Patients with EOC administratedby Department of Gynecologic Oncology, the affiliated Tumor Hospital of Harbin Medical University between August 2009 and April 2013 were prospectively recruited with informed consents. Plasma samples were obtained from patients prior to surgery. Samples were maintained at room temperature during transportation and then centrifuged at 1000 g for 10 min within four hours since collected and the isolated supernatant were extracted and stored at –80°C until further analysis. Patients enrolled in this study were not taking any medications and those suffering from metabolic diseases, liver diseases, kidney diseases or any other cancers were excluded. All the patients underwent complete cytoreductive surgery and received postoperative intravenous platinumbasedcombination chemotherapy. The interval of chemotherapy was 3 weeks. The chemotherapy regimen consisted of cisplatin plus paclitaxel or cisplatin, epirubicin and cyclophosphamide which was in line with National Comprehensive Cancer Network Guidelines.

### Follow-up and clinical endpoints

Following up was performed and disease recurrence or progression was recorded on routine hospital flow charts every 3 months for the first 1–2 yearafter surgery, and at 6-month intervals in 3–5 years. Patients were followed until recurrence or April, 2016. Due to patients recurring within the first 6 months are considered platinum resistant and require different therapeutic regimen, we defined patients who relapsed within the first 6 months after treatment as early relapse (ER). Patients relapsing after 6 months were included into the late relapse group (LR). No relapse group (NR) corresponded to patients showing no evidence of recurrence after at least 36 months of follow-up. Endpoint event was EOC recurrence. Recurrence was systematically assessed by conventional imaging (computed tomography), positron emission tomography (PET) scan or laparoscopic exploration.

### Sample preparation

All the plasma samples were thaw in 4°C and a 30 μl of plasma was mixed with 90 μl of precooled methanol. 300 ul methyl tert-butyl ether (MTBE) was then added into the mixture, which was oscillated at 1000 rpm in 25°C for 1 hour and was further added by 75 μl deionized water, vortex-mixed for 1 min, and oscillated at 1000 rpm in 4°C for 10 min, then the final mixture was centrifuged at 12000. 240 ul of upper layer was transferred into a clear vial and dried in vacuum rotary dryer. Lastly, the residue was dissolved in 100 μl of a 50/50 (v/v) solution of isopropanol/methonal for further analysis. To assess the stability and repeatability of the UPLC/MS systems, a total of 15 quality control (QC) samples were used in this study. The QC samples were prepared by pooling equal volumes of plasma from each of the 70 samples.

### Chromatography and mass spectrometry

A 5 μl aliquot of the pre-treated sample was injected a column of Kinetex Core-shell Silica C18 2.1 mm× 50 mm, 1.3 μl (Phenomenex, Torrance, CA, USA) on UPLC system (Waters, Milford, USA). The mobile phase consisted of acetonitrile/isopropanol 10/90 (v/v) (solvent A) and acetonitrile/deionized water 60/40 (v/v) (solvent B). The flow rate was set at 0.26 ml/min with a colume temperature of 40°C. A linear mobile phase gradient was used as follows: 10% A, held for 1 min; 1–8 min, increased to 30% A; 8–18 min, increased to 75% A; 18–20 min, increased to 97% A; 20–24 min, maintained at 97% A; 24–25 min, decreased to 10% A, and 25–26.4 min, maintained at 10% A. After each analytical running, the mobile phase was returned to 1% A for 0.1 min and equilibrated at 1% A for 1 min. To minimize the analytical variation, all samples were randomly analyzed in succession. Meanwhile, samples of quality control were analyzed at the beginning and the end of each running batch to ensure the stability during analysis.

Data acquisition were performed with an Agilent 6520-QTOF (Agilent Technologies) equipped with an electrospray ionization source operating at positive-ion electrospray ionization (ESI+) modes. The capillary voltage was 4.0 kV. Nitrogen was used as the dry gas, and the desolvation gas flow was set at 10 L/min. The desolvation temperature was set at 330°C. Centroid data were collected in the full scan mode from 50 to 1000 m/z.

### Data preprocessing and annotation

Raw data were converted into mzdata-format files by MassHunter Qualitative Analysis Software (Agilent Technologies) and then these files were imported to the XCMS package in R platform for preprocessing. The parameters were set as default values in XCMS function, following: xcmsSet (method=”centWave”, peakwidth5c (5, 20)); group (bw5); rector (method5”obiwarp”). The preprocessing results generated a data matrix that consisted of the retention time (RT), mass-to-charge ratio (m/z) values, and peak intensity. CAMERA package in R project was used for annotation of isotope peaks, adducts and fragments in the peak lists. Isotopic peaks were excluded prior to statistical analysis.

### Statistical analysis

Kruskal–Wallis rank sum test was used to determine the significance of each lipid (*P* < 0.05). Principal component analysis (PCA) was first used to detect stability and replication. Partial least squares discriminant analysis (PLS-DA) with mean centering and unit variance scaling of significant metabolites were carried out to understand global lipid changes between with and without EOC recurrence groups, as well as between ER and LR, NR. The parameters of the model, such as R2Y and Q2 were used to evaluate the over-fitting of model based on 7-fold cross-validation. The variable importance in the projection (VIP) values, calculated based on the established PLS-DA models with thresholds of 1.0, were used for the selection of potential biomarkers. Random forest model was used to evaluate predictive performance of potential biomarkers based on leave-one-out cross-validation in terms of area under the receiver operating characteristic (ROC). In terms of clinical applicability, we selected the lipid with strongest prediction performance that would be complementary to demographic and clinical predictors. The predictive accuracy of demographic and clinical predictors alone and with the predictive lipids was performed using the logistic regression modeling with AUC value. For both the potential lipid biomarkers and the clinical predictors, 2 models are presented. Model 1 includes clinical characteristics. Model 2 adds individual lipid. Statistical analysis was performed in the R platform, with the exception of PLS-DA which was analyzed using SIMCA-P (version 11.5; Umetrics, Malmo, Sweden).

## SUPPLEMENTARY MATERIALS FIGURES AND TABLES


