# Supplementary material for: Distinct plasma lipids profiles of recurrent ovarian cancer by liquid chromatography-mass spectrometry
Source: Oncotarget. 2016 Aug 25;8(29):46834–45. doi: 10.18632/oncotarget.11603 (PMC5564526; doi:10.18632/oncotarget.11603)
Supplement: Supplementary file 1 [file oncotarget-08-46834-s001.pdf]

Distinct plasma lipids profiles of recurrent ovarian cancer by liquid chromatography-mass spectrometry

Supplementary Materials

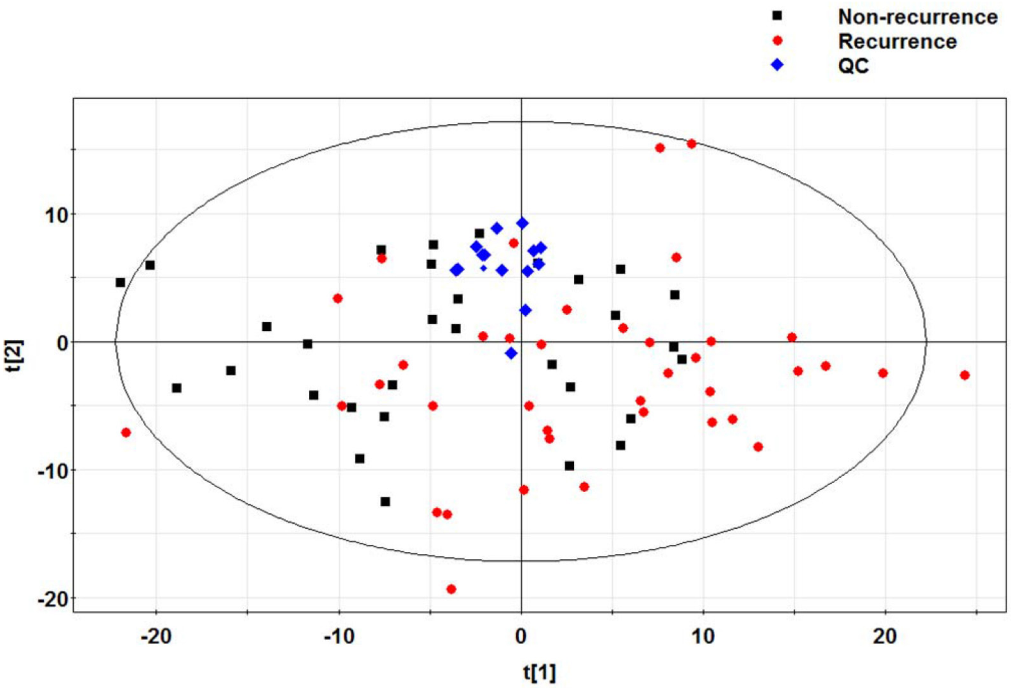

Supplementary Figure S1: PCA score plot for discriminating recurrent EOC, non-recurrent EOC and QC (quality control).

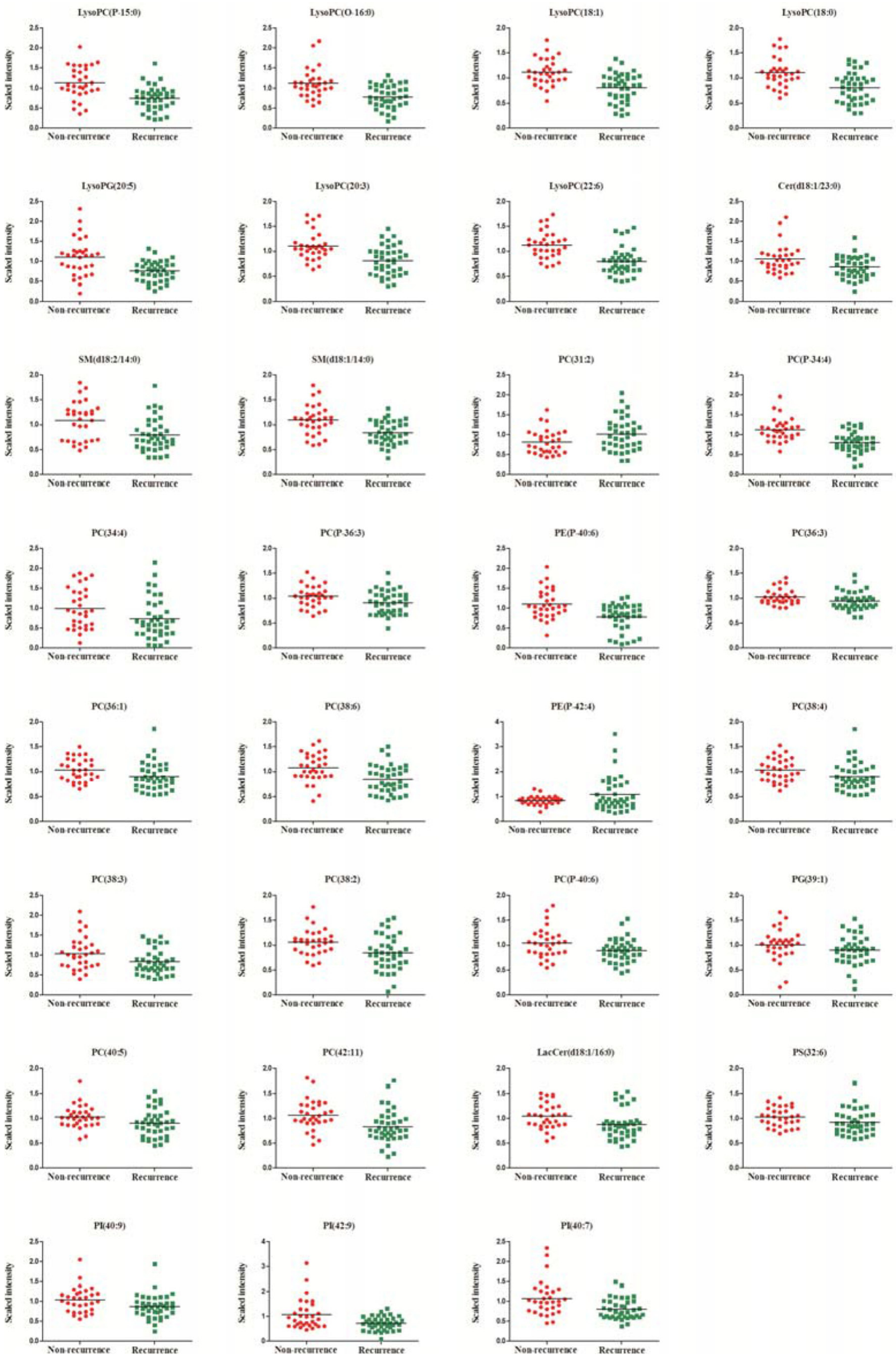

Supplementary Figure S2: Individual data for the concentrations of thirty-one lipids.

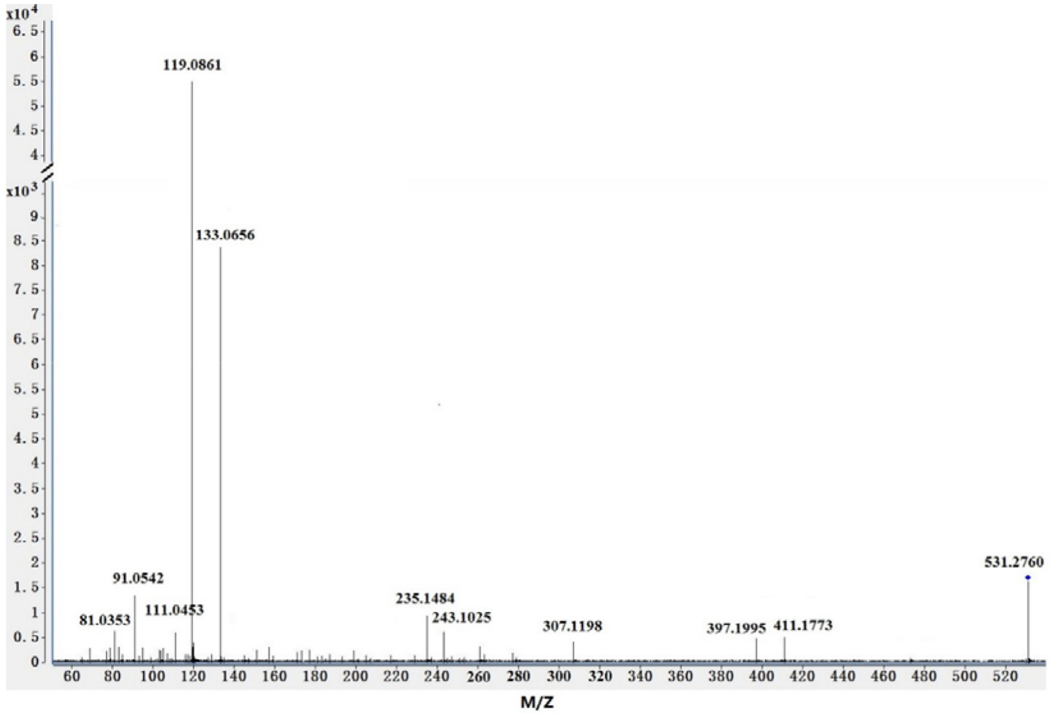

Supplementary Figure S3: Mass spectra for LysoPG (20:5).

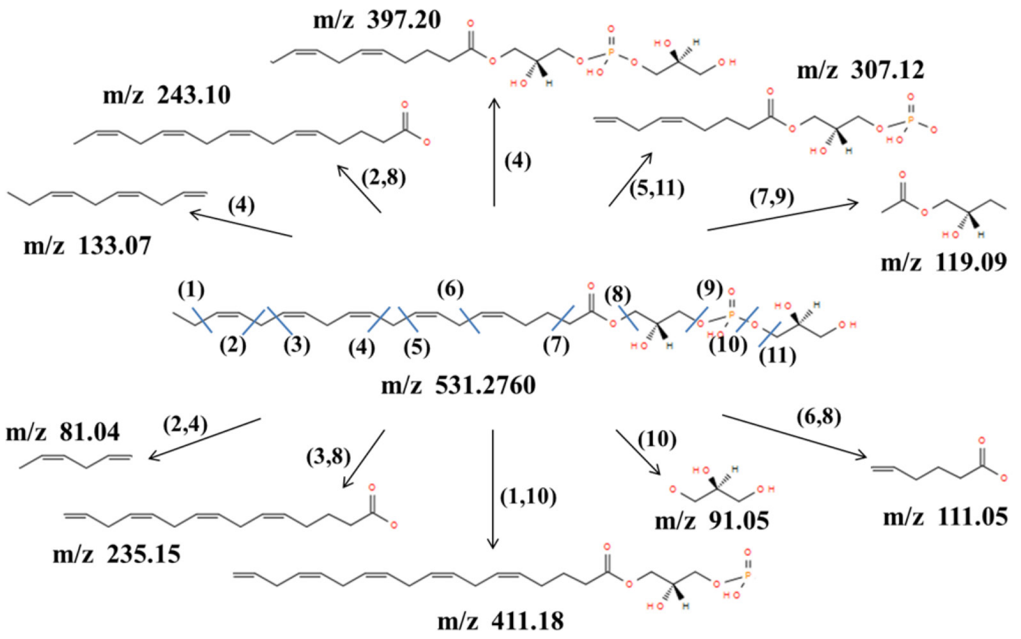

Supplementary Figure S4: The possible fragment structures of LysoPG(20:5).

Supplementary Table S1: Univariate COX regression analysis for the association of PFS with demographic and clinical characteristics between recurrent and non-recurrent EOC patients

Parameter	Estimate	SE	Chi-Square	*P* value	HR	95%CI	
						lower	Upper
Age	0.0084	0.0161	0.2700	0.6093	1.008	0.977	1.041
CA125	1.7049	1.0138	2.8279	0.0926	5.501	0.754	40.122
greater omentum metastasis	1.3345	0.4053	10.8391	0.0010	3.798	1.716	8.406
FIGO stage	0.9273	0.2915	10.1030	0.0015	2.528	1.427	4.478
Histology differentiation	1.0023	0.3868	8.7159	0.0096	2.725	1.277	5.815
Lymph node metastasis	1.0626	0.3398	9.7826	0.0018	2.894	1.487	5.632
LysoGP(20:5)	–2.0668	0.6036	11.7231	0.0006	0.127	0.039	0.413
